# Ten years of DuoStim in time-sensitive poor-prognosis IVF patients: real world cumulative outcomes and treatment trajectories

**DOI:** 10.3389/fendo.2026.1854570

**Published:** 2026-08-18

**Authors:** Filippo Maria Ubaldi, Federica Innocenti, Marilena Taggi, Silvia Colamaria, Cindy Argento, Maddalena Giuliani, Alessandro Ruffa, Erika Pittana, Chiara Gallo, Susanna Ferrero, Michele Morelli, Maurizio Guido, Laura Rienzi, Danilo Cimadomo, Alberto Vaiarelli

**Affiliations:** 1 Genera, Clinica Valle Giulia, IVIRMA Global Research Alliance, Rome, Italy; 2 Department of Pharmacy, Health and Nutritional Sciences, University of Calabria, Rende, Italy; 3 IVIRMA Italia, IVIRMA Global Research Alliance, Rome, Italy; 4 Department of Obstetrics and Gynaecology, University Magna Graecia, Catanzaro, Italy; 5 Department of Biomolecular Sciences, University of Urbino “Carlo Bo”, Urbino, Italy; 6 Department of Biology and Biotechnology “Lazzaro Spallanzani”, University of Pavia, Pavia, Italy

**Keywords:** duostim, ovarian stimulation, time to live birth, time to treatment conclusion, treatment discontinuation

## Abstract

**Aims:**

To evaluate DuoStim strategy across a decade from its clinical implementation in IVF by comparing embryological and clinical outcomes between the first and second ovarian stimulations (1st-OS and 2nd-OS) conducted within 30 days, focusing on treatment efficiency and time-to-conclusion.

**Methods:**

Retrospective single-center study (2015–2024) including 1656 poor-prognosis patients undergoing 1896 DuoStim cycles planned for ICSI and PGT-A at the blastocyst stage. Poor prognosis was defined as very advanced maternal age (≥40 years) and/or reduced ovarian reserve (AMH ≤1.2 ng/mL, AFC ≤5, or ≤3 oocytes in previous retrievals). Endpoints included all embryological, clinical and neonatal outcomes comparing 2nd-OS versus 1st-OS and cumulatively. Among 1561 concluded first DuoStim cycles, we calculated time-to-treatment-conclusion (TTC), stratified as without a live-birth (TTC-noLB) and with a live-birth (TTC-LB), and treatment discontinuation at our center after a first failed DuoStim attempt.

**Results:**

The use of DuoStim increased over time, reaching 34% of all cycles in 2024. The 2nd-OS yielded more oocytes and embryos than 1st-OS, while their competence was comparable. Consequently, the 2nd retrieval provided a cumulative incremental yield of 66% for cycles achieving ≥1 euploid blastocyst and 125% for cycles achieving ≥1 live birth (LB) compared with the 1st-OS alone. Median TTC was 40 days (quartile-1: 36, quartile-3: 118.5), while TTC-noLB and TTC-LB were 38 days (quartile-1: 35, quartile-3: 41.5) and 145 days (quartile-1: 112.25, quartile-3: 221), respectively. Embryological and clinical outcomes were stable across the years. Treatment discontinuation among patients without a LB after a first completed DuoStim decreased from 61% in 2015 to 44% in 2021, as second DuoStim attempts increased from 16% to 21% and transition to egg donation from 9% to 21%.

**Conclusion:**

DuoStim is a multicycle stimulation strategy that represents an alternative approach for patients requiring multiple treatment opportunities within a short timeframe. It shifts IVF toward a flexible personalized journey for poor prognosis patients. By enabling two stimulations within a single ovarian cycle, it achieved a cumulative live birth rate of 28.5% per concluded cycle in our cohort, within a consistently short timeframe in terms of both TTC-noLB and TTC-LB. Overall, DuoStim represents a treatment strategy that supports rapid and effective clinical decision-making in time-sensitive patients.

## Introduction

Over the past decades, a progressive decline in fertility has been observed worldwide, largely reflecting delayed childbearing driven by social and economic factors and the well-established age-related decline in female reproductive potential (1). Consequently, an increasing number of patients seeking assisted reproductive technology (ART) approach this journey at a more advanced reproductive age, often characterized by diminished ovarian reserve and narrower reproductive windows. Thus, ART has evolved into a new paradigm where success is no longer defined by the outcome of a single embryo transfer or an ovarian stimulation cycle, but a cumulative approach across multiple attempts is required. From a biological perspective, ovarian stimulation (OS) cannot generate new oocytes, but only recruit follicles already present within the ovary. In fact, the number of oocytes retrieved represents the main limiting factor to cumulative success, directly influencing the number of embryos available for transfer and the overall probability of a live birth (LB) per initiated treatment (2). Recent advances in ovarian physiology have demonstrated that multiple waves of follicular recruitment arise within the same menstrual cycle, rather than a single wave as traditionally postulated (3, 4). This revised understanding of ovarian dynamics paved the way to more flexible unconventional stimulation strategies like random-start and luteal-phase stimulation, that allow OS to be initiated at different time points during the cycle (5). Within this context, DuoStim was designed as two consecutive OS performed back-to-back within the same ovarian cycle, embodying the clinical application of these physiological insights. Its early conceptualization was grounded by a translational process integrating experimental observations from animal models with clinical evidence initially produced by Kuang and colleagues. First described in 2014, the so-called “Shanghai protocol”, was proposed to increase oocyte yield in poor responder patients (6), and provided proof that two consecutive OS can be safely and effectively performed within a single ovarian cycle, thereby maximizing oocyte yield within a limited timeframe (7). Over time, its clinical indications have expanded to include a broader group of so-called “time-sensitive” patients, such as women requiring urgent fertility preservation, very advanced maternal age women and/or patients with reduced ovarian reserve (2, 3, 8–10). Through the years, mounting evidence highlighted that DuoStim leads to a relevant increase in the total number of oocytes and embryos compared with conventional OS, without compromising oocyte competence (2–4). Notably, the oocytes collected after the second OS (2nd-OS) were more numerous than after the first (1st-OS), possibly due to a priming effect deriving from the latter (5), and suggesting a potential improvement in treatment efficiency and cost-effectiveness (8–10). All these findings contributed to the evidence base summarized in systematic reviews and meta-analyses, which overall support the safety, reproducibility, and biological plausibility of DuoStim (11, 12). Recent studies suggested that DuoStim may also reduce the time required to obtain a euploid embryo while maintaining comparable outcomes for other parameters (13, 14). However, some randomized trials have reported less consistent benefits (15, 16). In fact, some limitations associated with DuoStim should be acknowledged. Its application is primarily restricted to selected patient populations, namely those with poor prognosis or time-sensitive reproductive needs, and its benefits cannot be extended to unselected IVF cohorts. Although the protocol is biologically feasible, its implementation requires a high level of expertise, which may limit its reproducibility across centers. Heterogeneity across studies, particularly regarding oocyte handling, embryo culture, and transfer policies complicates direct comparison of outcomes and may contribute to variability in effectiveness. Therefore, DuoStim value is largely grounded on cumulative oocyte and embryo yield within a compressed timeframe, rather than improvements in per-cycle efficiency, which introduces conceptual challenges when comparing it to conventional OS strategies.

Over time, the clinical application of DuoStim has been further refined through an increasing multicenter experience and the inherent flexibility of the protocol, including the possibility of suggesting this strategy during the treatment based on the embryological outcomes of an ongoing attempt. From a health economics perspective, its value is supported by data suggesting efficient resource allocation within complex IVF pathways, particularly in cases that might benefit from oocyte/embryo accumulation (17, 18). In addition, follow-up data on offspring provide reassuring evidence regarding neonatal and perinatal outcomes, supporting the safety of the approach in routine clinical practice (19). Importantly, DuoStim should not be regarded as a stand-alone intervention, but rather integrated within a broader, personalized multicycle OS strategy, where treatment intensity and scheduling are tailored to the patient’s reproductive profile, prognosis, and time constraints.

Ten years after the implementation of DuoStim at our center, a comprehensive evaluation of its clinical effectiveness in routine practice was warranted. This observational study was designed to describe the evolution of DuoStim adoption in our clinical setting, defining treatment success within a multicycle framework, reporting cumulative outcomes, time metrics and treatment journeys after a first failed DuoStim attempt.

## Methods

### Study design, patient population and outcome measures

This retrospective single-center observational study was conducted at a private IVF center in Rome between 2015 and 2024 and included 1656 patients who underwent ≥1 DuoStim cycle (**Supplementary Figure S1** represents a detailed summary of all study outcomes). A total of 1896 DuoStim cycles were performed, of which 1776 (94%) were concluded either with a LB achieved [defined according to (20, 21)] or with no surplus euploid blastocyst available for transfer (1760 1st-OS and 1756 2nd-OS were respectively concluded). All patients were classified as poor-prognosis, defined by one or more of the following criteria: maternal age ≥40 years, anti-Müllerian hormone (AMH) ≤1.2 ng/mL, antral follicle count (AFC) ≤5, or previous oocyte retrievals yielding ≤3 oocytes (22–24).

Firstly, we reported the annual prevalence of DuoStim, defined as the yearly proportion of DuoStim cycles relative to the total number of oocyte retrievals conducted at the center, to describe the progressive adoption of this strategy over time and the volume of patients managed with this approach. Secondly, we conducted a detailed comparative analysis of embryological and clinical outcomes between the 1st-OS and the 2nd-OS, including a descriptive analysis of the incremental proportion of patients with ≥1 euploid embryo and ≥1 LB. For each oocyte pick up (OPU), we evaluated the mean number of cumulus-oocyte-complexes (COCs), metaphase II oocytes (MIIs), normally fertilized oocytes with two pronuclei (2PNs), blastocysts and euploid. Also, maturation, fertilization, blastulation, and euploid blastocyst rates were calculated and compared. Positive pregnancy test rate per transfer, biochemical pregnancy loss rate per positive pregnancy test, miscarriage rate per clinical pregnancy and live birth rate (LBR) per transfer were also reported. Thirdly, neonatal outcomes were assessed and compared between 1st-OS and 2nd-OS, including gestational age, birthweight, and malformations to further characterize the overall safety profile of the treatment. This information is generally collected through phone interviews and was available for a subset of all deliveries achieved in the study period (N = 224/533, 42%). Neonatal data were analysed using a complete-case approach, namely including only cases with available follow-up information. Preterm delivery, low birthweight, small and large for gestational age were all defined according to the World Health Organization (25, 26). Fourthly, time-to-treatment-conclusion (TTC) was calculated for the first DuoStim cycle for each couple. TTC was defined as the number of days between the initiation of the 1st-OS and the last practice performed in the clinic that determined the conclusion of the treatment either with or without a LB. Therefore, TTC was sub-categorized as time to no LB (TTC-noLB) and time to LB (TTC-LB). The former included absence of oocyte retrieved, of mature oocytes, of normally fertilized zygotes, absence of blastocysts, of euploid blastocysts, and of LB after euploid transfer (including implantation failure, biochemical pregnancy loss, and clinical miscarriage). TTC-LB, instead, corresponded to the number of days from the start of 1st-OS to the date of embryo transfer that resulted in the LB. Lastly, treatment discontinuation after a first failed DuoStim cycle was evaluated. Discontinuation was operationally defined as the absence of subsequent ART cycles (including additional DuoStim cycles, conventional OS, or transition to oocyte donation) at our center following the first DuoStim. This analysis was restricted to patients who initiated their first DuoStim between 2015 and 2021, allowing for up to 3 years of observation after the start of the 1st-OS. Patients who did not return for further attempts within this period were classified as having discontinued treatment at our center, although they might have started a new treatment at another center or conceived spontaneously. All study outcomes were analysed throughout the study period to describe temporal trends.

### Ovarian stimulation and IVF procedures

Each DuoStim cycle consisted of two consecutive OS performed within a single ovarian cycle. In the initial implementation of the DuoStim strategy, ovarian stimulation was carried out using gonadotropins in combination with a flexible GnRH antagonist protocol to prevent a premature LH surge. Before the initiation of stimulation, patients underwent transvaginal ultrasound and baseline ovarian assessment on day 2–3 of the menstrual cycle. A dose of recombinant or urinary gonadotropins (225–300 IU/day) was administered. The GnRH antagonist was introduced when the leading follicle reached approximately 13–14 mm in diameter. Follicular development was monitored by transvaginal ultrasound starting from day 5 of stimulation until the day of triggering. Final oocyte maturation was induced with a GnRH agonist when at least three follicles reached a mean diameter ≥16 mm. More recently, this protocol has evolved with the introduction of progestin-primed ovarian stimulation (PPOS) (27, 28). In this approach, oral progesterone is administered during ovarian stimulation instead of daily GnRH antagonist injections to prevent a premature LH surge. This modification simplifies the treatment regimen and reduces the number of injections while maintaining comparable clinical outcomes (29). COCs were retrieved 35–36 hours after ovulation trigger via transvaginal ultrasound-guided aspiration of follicular fluid. Oocytes were then denuded in a HEPES-buffered medium and inseminated by ICSI as described previously (30, 31). Fertilization was evaluated 16–17 hours post-ICSI based on the presence of two equally sized pronuclei. Embryos were cultured individually in a controlled humidified atmosphere (37°C, 6% CO_2_, 5% O_2_) until full blastocyst expansion on day 5–7, at which point trophectoderm biopsy was performed for PGT-A following a protocol that did not involve day 3 zona pellucida drilling (32, 33). Comprehensive chromosome analysis was conducted via qPCR (until October 2017) or NGS at an external laboratory. All embryo transfers consisted of a single euploid blastocyst following warming.

### Endometrial preparation and euploid embryo transfer

Endometrial preparation and embryo transfer procedures have been described previously (34, 35). Briefly, only single euploid blastocyst transfers were performed in either a hormone replacement therapy (HRT) cycle or a natural/modified natural cycle. Given the hormonal milieu associated with double ovarian stimulation within the same menstrual cycle, a freeze-all strategy was adopted. Embryo transfer was carried out under transabdominal ultrasound guidance using a soft catheter. Pregnancy testing was conducted 11 days after the transfer, followed by clinical pregnancy confirmation via ultrasound at 5–6 weeks’ gestation.

### Statistical analyses

Statistical analysis was performed using SPSS version 29 (IBM, USA). The distribution of continuous variables was assessed using the Shapiro–Wilk test. Continuous variables are presented as median with first and third quartiles (Q1–Q3), while descriptive means and standard deviations (SD) are additionally reported for descriptive purposes. Categorical variables are presented as counts and percentages. For embryological outcomes between 1st-OS and 2nd-OS, absolute numbers (COCs, MII oocytes, 2PNs, blastocysts and euploid blastocysts) were compared using the Wilcoxon signed-rank test, rates, instead, were compared using the Mann–Whitney U test except for except for the mean euploid blastocyst rate per COC. Effect estimates were reported as Hodges–Lehmann median differences with corresponding 95% confidence intervals. Categorical variables were compared using the χ² test or Fisher’s exact test, as appropriate. Comparisons across years were performed using one-way analysis of variance (ANOVA) for normally distributed variables or the Kruskal–Wallis test for non-normally distributed variables. Univariate logistic regression analyses were initially performed to evaluate the association between treatment discontinuation after a first failed DuoStim cycle and the following variables: year of treatment (categorical), maternal age (continuous), number of COCs retrieved after the 1st-OS (continuous), number of previous cycles performed (continuous), presence of severe male factor infertility (oligoasthenoteratozoospermia or azoospermia; yes/no), main cause of female infertility (categorical variable including idiopathic, diminished ovarian reserve, advanced maternal age, endocrine-ovulatory disorders, endometriosis, and tubal factor), duration of infertility (continuous), and TTC of the first DuoStim cycle (continuous). Variables showing significant associations in the univariate analyses were further evaluated in a multivariate logistic regression model to confirm independent associations. Potential collinearity among variables was assessed based on variance inflation factor (VIF). The events per variable (EPV) ratio was reported based on the number of multivariate model parameters to ensure model stability and minimize the risk of overfitting. Model discrimination was assessed using the area under the receiver operating characteristic curve (AUC). Logistic regression analyses with a generalized estimating equations (GEE) approach were also performed to outline associations with clinical and neonatal outcomes after single euploid blastocyst transfers (SEBT), while accounting for repeated embryo transfers and LBs from the same couple. A p-value ≤ 0.05 was considered statistically significant.

## Results

### Annual prevalence of DuoStim cycles

**Figure 1:**
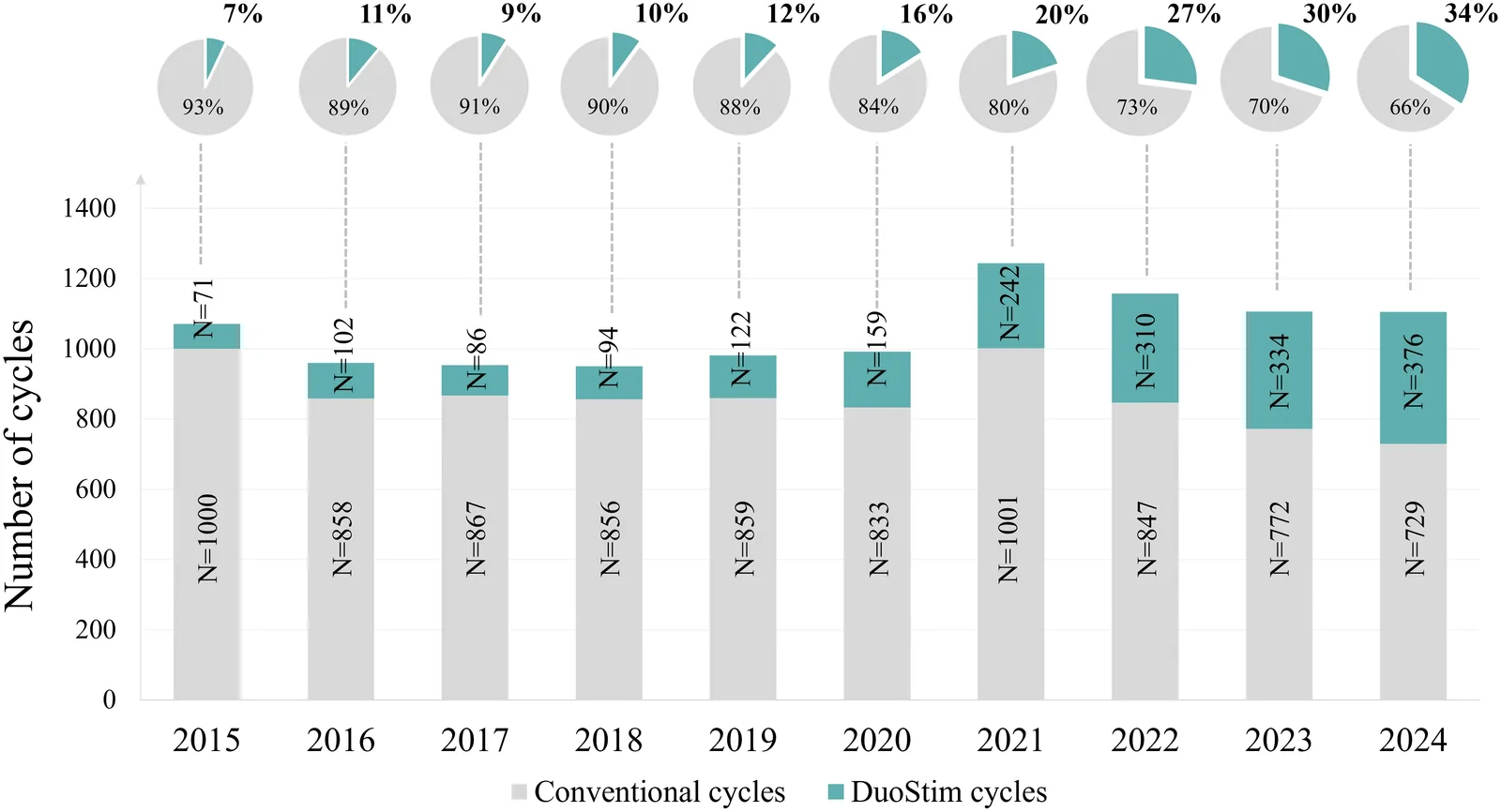
Longitudinal trends in the use of DuoStim strategy. Annual distribution of ovarian stimulation cycles performed between 2015 and 2024, showing the progressive increase in the proportion of DuoStim cycles (green) relative to conventional ovarian stimulation cycles (grey). The corresponding percentages are reported above each year.

**Figure 2:**
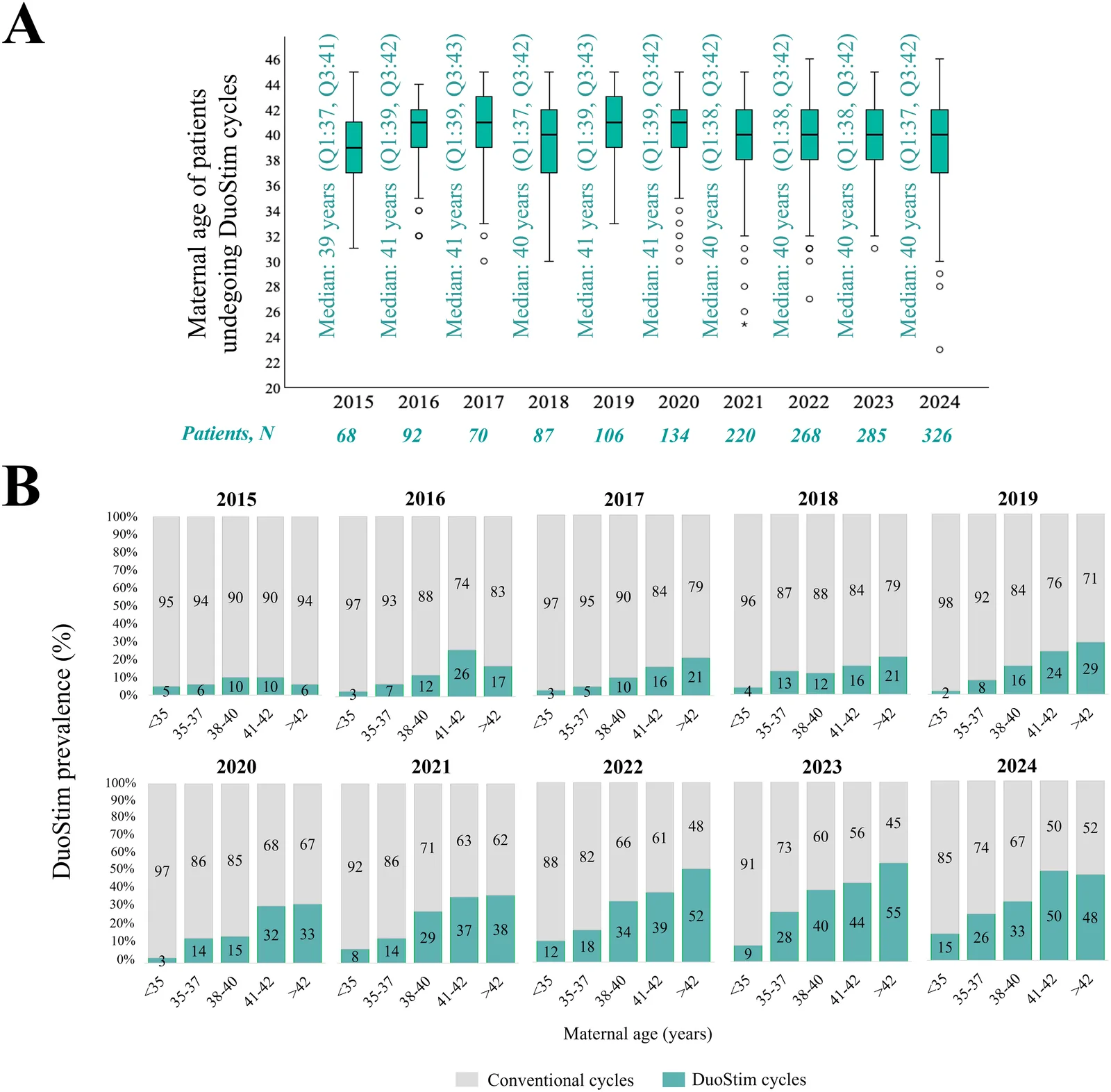
Maternal age distribution. **(A)** Median maternal age of patients undergoing DuoStim cycles from 2015 to 2024. **(B)** Percentage distribution of patients across five age groups (≤ 35, 35-37, 38-40, 41-42, and > 42 years) comparing conventional and DuoStim cycles.

Between 2015 and 2024, a total of 1896 DuoStim cycles were performed, with a progressive increase in their annual prevalence relative to overall activity, rising from 6.6% in 2015 to 34% in 2024 (**Figure 1**). Baseline characteristics of patients undergoing DuoStim versus conventional approaches are summarized in **Table 1**. As expected, DuoStim patients were older and exhibited a distinct hormonal profile, with higher FSH and lower LH and AMH levels, consistent with the application of this strategy in poor-prognosis patients. As shown in **Figure 2A**, maternal age among patients undergoing DuoStim remained stable throughout the study period, with a median consistently around 39–40 years and limited variability, indicating a relatively homogeneous population of advanced reproductive age. **Figure 2B** further illustrates the distribution of patients across five age groups (≤ 35, 35-37, 38-40, 41-42, and > 42 years), comparing conventional and DuoStim cycles. A progressive increase in the proportion of DuoStim cycles was observed across all age categories, accompanied by a relative decline in conventional cycles. This trend became more pronounced in the most recent years (2021–2024), particularly in older age groups. Overall, these findings demonstrate a growing adoption of the DuoStim approach in clinical practice across all age ranges, while remaining predominantly utilized in patients around 40 years of age.

**Table 1:** Description of patient populations undergoing conventional ovarian stimulation and DuoStim cycles (2015-2024).

Parameters	Conventional-OS (N=6124)	DuoStim (N=1656)	*P*-value
**Maternal age** (median; Q1-Q3)	38 (35-41) years	40 (38-42) years	<0.001
**Paternal age** (median; Q1-Q3)	40 (37-44) years	42 (38-45) years	<0.001
**Female BMI** (median; Q1-Q3)	21.4 (19.7-23.6) kg/m²	21.5 (19.73-23.70) kg/m²	0.797
**FSH** (median; Q1-Q3)	7.4 (5.8-9.76) mIU/ml	8 (6.2-11) mIU/ml	<0.001
**LH** (median; Q1-Q3)	5.6 (4.06-7.6) mIU/ml	5.3 (3.8-7.21) mIU/ml	<0.001
**AMH** (median; Q1-Q3)	1.75 (0.87-3.3) mIU/ml	1 (0.5-1.8) mIU/ml	<0.001
Type of infertility (n/N; %)			
Primary	N=4670/6124; 76.3%	N=1254/1656; 75.7%	0.338
Secondary	N=1454/6124; 23.7%	N=402/1656; 24.3%	
Main cause of female infertility (n/N; %)			
Idiopathic	N=915/6124; 14.9%	N=53/1656; 3.2%	<0.001
DOR	N=132/6124; 2.2%	N=33/1656; 2.0%	
AMA	N=4019/6124; 65.6%	N=1416/1656; 85.5%	
Endocrine-ovulatory	N=289/6124; 4.7%	N=23/1656; 1.4%	
Endometriosis	N=333/6124; 5.4%	N=79/1656; 4.8%	
Tubal factor	N=436/6124; 7.1%	N=52/1656; 3.2%	
Severe male factor infertility (n/N; %)			
Absent	N=4987/6124; 81.4%	N=1437/1656; 86.8%	<0.001
Present	N=1137/6124; 18.6%	N=219/1656; 13.2%	
**Years of infertility,** (median; Q1-Q3)	2 (1-4) years	2 (1-3) years	0.460

OS, ovarian stimulation; BMI, Body Mass Index; FSH, Follicle-Stimulating Hormone; LH, Luteinizing Hormone; AMH, Anti-Müllerian Hormone; DOR, Diminished Ovarian Reserve; AMA, Advanced Maternal Age; Q1, quartile 1; Q3, quartile 3.

### Embryological outcomes following 1st-OS vs 2nd-OS

Among 1896 DuoStim cycles, 2nd-OS yielded more COCs with an estimated median difference of 1 COC per OPU (95% CI: 0.5–1). Similarly, more MII oocytes (median difference 0.5, 95% CI: 0.5–1) and 2PN zygotes (median difference 0.5, 95% CI: 0.5–0.5) were also obtained per OPU following the 2nd-OS. A significant, although negligible, difference in maturation rate was observed between the two OS, while fertilization rates were comparable. The number of blastocysts obtained per OPU was also higher following the 2nd-OS (median difference 0.5, 95% CI: 0.5–0.5), whereas blastulation rates and euploidy rates per biopsied blastocyst did not differ. The overall oocyte competence defined as mean euploid blastocyst rate per COC was similar in the two OS (**Table 2** summarizes all embryological outcomes). These data were consistent throughout the study period.

**Table 2:** Embryological outcomes in DuoStim cycles: comparison between 1st-OS and 2nd-OS.

Embryological outcome	1st-OS	2nd-OS	Hodges-Lehman median difference with 95%CI p-value
COCs per OPU (median, Q1 – Q3) (descriptive mean ± SD)	5, 3-8 5.9±3.9	6, 4-9 6.8±4.2	1 (0.5-1) <0.001
MIIs per OPU (median, Q1 – Q3) (descriptive mean ± SD)	3, 2-5 4.0±3.0	4, 2-7 4.8±3.4	0.5 (0.5-1) <0.001
Mean Maturation rate (median, Q1 – Q3) (descriptive mean ± SD)	75%, 50-91 69.2±26.2%	75%, 57-90 71.5±24.0%	0 (0-0) 0.036
2PNs per OPU (median, Q1 – Q3) (descriptive mean ± SD)	2, 1-4 2.8±2.4	3, 1-5 3.5±2.8	0.5 (0.5-0.5) <0.001
Mean Fertilization rate (median, Q1 – Q3) (descriptive mean ± SD)	75%, 50-100 70.5±31.1%	75%, 56-100 71.8±28.7%	0 (0-0) 0.698
Blastocysts per OPU (median, Q1 – Q3) (descriptive mean ± SD)	1, 0-2 1.3±1.3	1, 0-3 1.7±1.6	0.5 (0.5-0.5) <0.001
Mean Blastulation rate (median, Q1 – Q3) (descriptive mean ± SD)	50%, 14-80 48.1±36.7%	50%, 20-75 48.9±35.1%	0 (0-0) 0.410
Euploid blastocysts per OPU (median, Q1 – Q3) (descriptive mean ± SD)	0, 0-1 0.6±0.8	0, 0-1 0.7±1	0 (0-0) <0.001
Mean Euploidy rate per biopsied blastocyst (median, Q1 – Q3) (descriptive mean ± SD)	0%, 0-50 30.9±39.4%	0%, 0-50 29.6±36.6%	0 (0-0) 0.863
Mean Euploid blastocyst rate per COC (median, Q1 – Q3) (descriptive mean ± SD)	0%, 0-11 7.1±14.1%	0%, 0-13 7.7±13.8%	0 (0-0) 0.059

COCs, cumulus oocyte complexes; OPU, ovum-pick up; MII, metaphase-II; 2PN, 2 pronuclei; Q1, quartile 1; Q3, quartile 3.

Wilcoxon Signed Rank paired tests for absolute numbers and Mann Whitney U tests for rates were adopted, except for the Mean Euploid blastocyst rate per COC..

### The outcomes after euploid single embryo transfer were comparable

**Figure 3:**
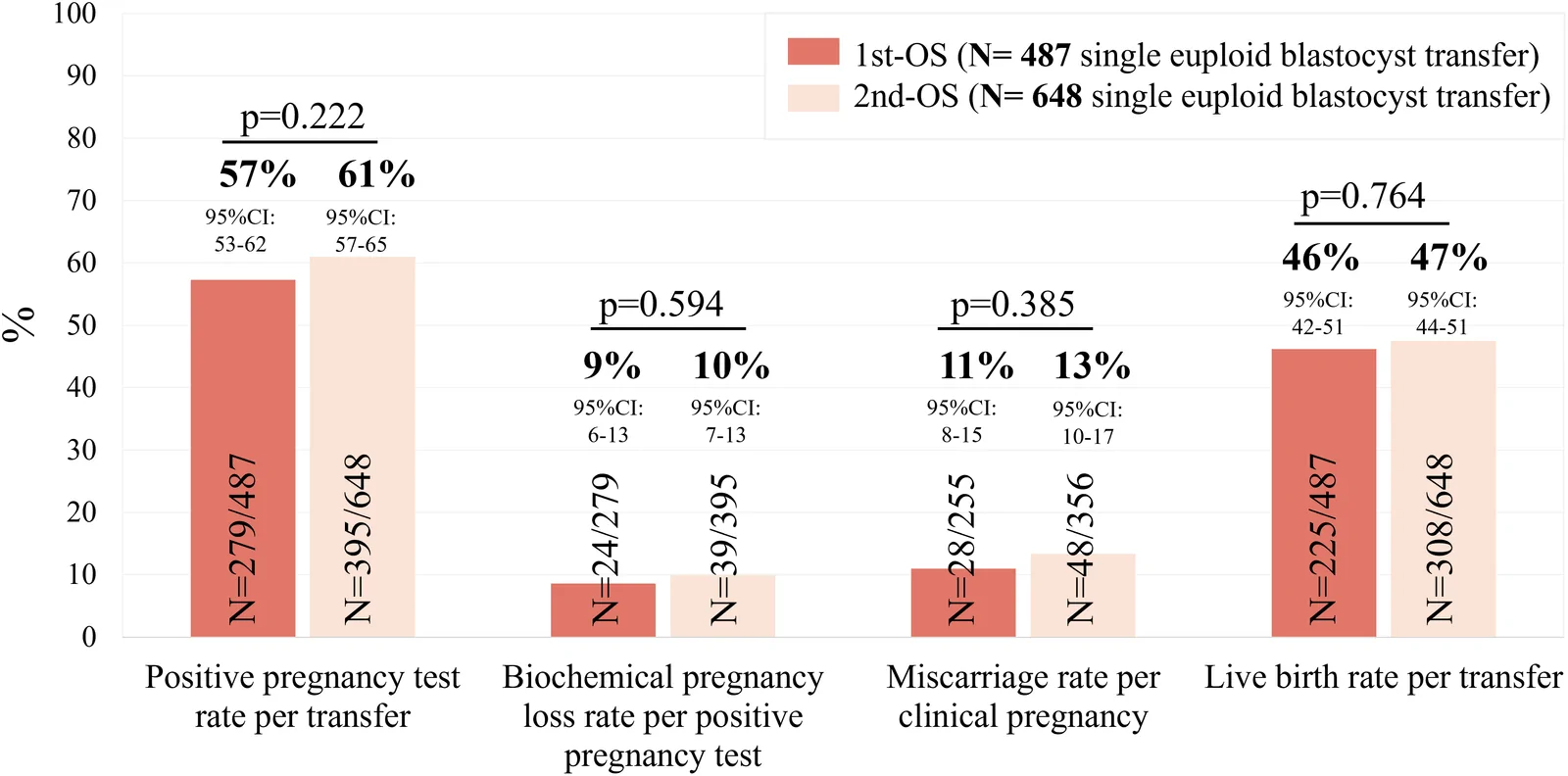
Clinical outcomes following single euploid blastocyst transfer after 1st-OS and 2nd-OS within DuoStim cycles. Rates of positive pregnancy test, biochemical pregnancy loss after a positive test, miscarriage following clinical pregnancy, and live birth (LB) per transfer are shown for 1st-OS (dark red, N = 487) and 2nd-OS (light red, N = 648). No statistically significant differences were observed between the two groups.

All clinical outcomes following SEBT were evaluated by comparing the 1st-OS and 2nd-OS. Overall, 1135 euploid SEBTs were analyzed, including 487 derived from the 1st-OS and performed in 440 patients, and 648 derived from the 2nd-OS and performed in 543 patients. No difference was reported and the LBR per transfer was 46% (N = 225/487; 95%CI 41.8% to 50.6%) after the 1st-OS and 47% (N = 308/648; 95%CI 43.7% to 51.4%) after the 2nd-OS, respectively (p=0.764) (**Figure 3**).

The data were reproducible throughout the study period and adjusting for multiple ETs conducted from the same patient with a GEE approach (OR of the LBR per transfer in 2nd-OS vs 1st-OS: 1.038, 95%CI 0.82-1.32, p = 0.760).

### Comparison of neonatal outcomes between 1st-OS and 2nd-OS

**Figure 4:**
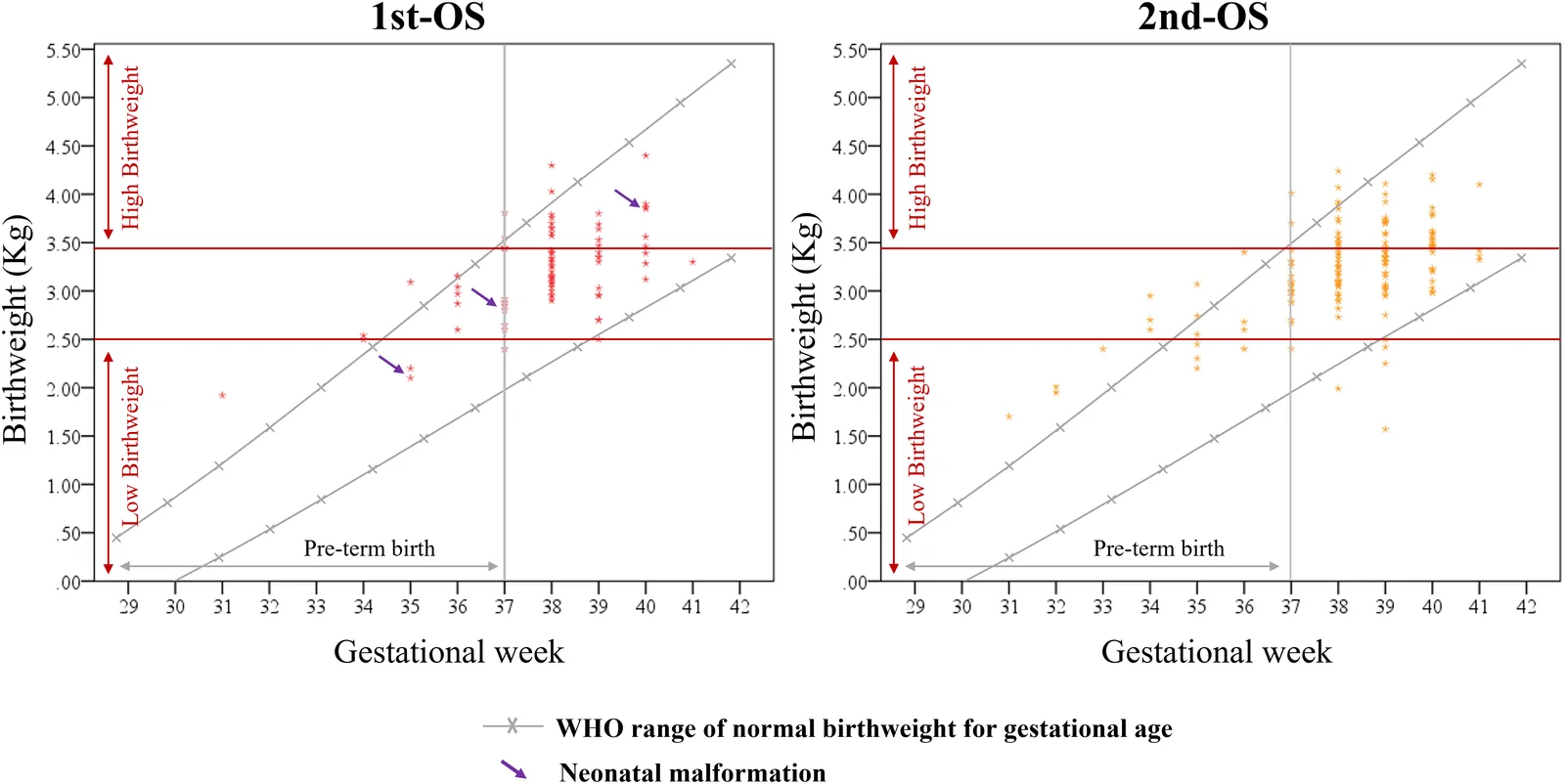
Neonatal outcomes following single euploid blastocyst transfer after 1st-OS and 2nd-OS within DuoStim cycles. Dispersion plot displaying each newborn (dark orange stars, 1st-OS-derived newborns; light orange stars, 2nd-OS-derived newborns) according to the gestational week and birthweight. The newborns suffering from a neonatal malformation are highlighted with a violet arrow. The ranges of birthweight per gestational age are displayed according to the World Health Organization (WHO) as grey lines. The grey double-headed arrow sets the threshold for pre-term birth. The red double-headed arrows set the threshold for low (lower line) and high (upper line) birthweight.

Neonatal outcomes were available for a subset of LBs (N = 224). **Figure 4** summarizes these outcomes according to stimulation cycle (1st-OS vs 2nd-OS). Preterm delivery rates (<37 weeks) were 15% (N = 12/79; 95%CI 8.9% to 24.7%) in the 1st-OS group and 12% (N = 17/145; 95%CI 7.4% to 18%) in the 2nd-OS group (p=0.53). Regarding birthweight, N = 1/79 (1.3%; 95%CI 0.2% to 6.8%) newborns in the 1st-OS group were small for gestational age (SGA), N = 73/79 (92.4%; 95%CI 84.4% to 96.5%) were normal for gestational age (NGA), and N = 5/79 (6.3%; 95%CI 2.7% to 14%) were large for gestational age (LGA). In the 2nd-OS group, these data were N = 5/145 (3.4%; 95%CI 1.5% to 7.8%), N = 129/145 (89%; 95%CI 82.8% to 93.1%), and N = 11/145 (7.6%; 95%CI 4.3% to 13.1%), respectively. The distributions were similar between groups (p=0.610). The same results were confirmed when adjusting for multiple LBs from the same patient with a GEE approach. Regarding malformations, N = 3/79 (3.8%; 95%CI 1.3% to 10.6%) newborns in the 1st-OS group presented with congenital anomalies. These included cerebral ventricular dilation, hand polydactyly, and unilateral renal agenesis. In the 2nd-OS group, none was reported. Given the small number of events, these analyses should be interpreted as exploratory.

### Incremental cLBR of 2nd-OS in the same ovarian cycle

**Figure 5:**
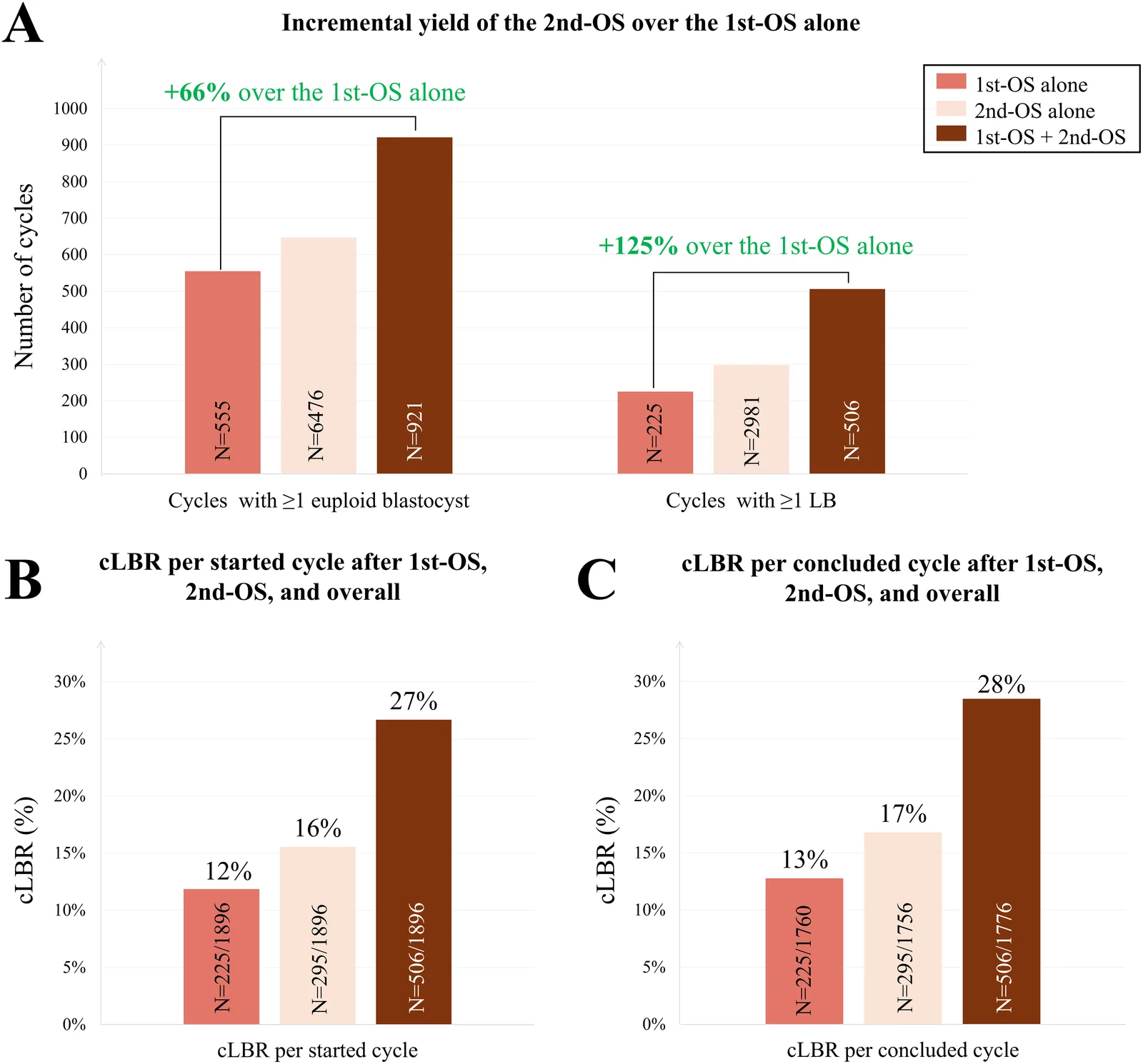
Incremental contribution to cumulative embryological and clinical outcomes of the second ovarian stimulation (2nd-OS) within DuoStim cycles over the 1st-OS alone. Figure **(A)** illustrates the number of cycles obtaining ≥1 euploid blastocyst and ≥1 live birth (LB) in the 1st-OS alone, in the 2nd-OS alone and combining them. The incremental yield of the 2nd-OS over the 1st-OS is also reported. Figure **(B)** illustrates the cumulative live birth rate (cLBR) per started cycle, while Figure **(C)** reports the cLBR per concluded cycle, both in the 1st-OS, in the 2nd-OS, and combining them. The three denominators (1760, 1756 and 1776) reflect the number of concluded cycles for each OS and overall, respectively.

To quantify the incremental yield contributed by the 2nd-OS in the same ovarian cycle, we assessed the additional number of cycles achieving ≥1 euploid blastocyst and ≥1 LB after the 2nd-OS who did not achieve these outcomes after the 1st-OS alone. Specifically, the number of cycles achieving ≥1 euploid blastocyst increased from 555 after the 1st-OS alone to 921 after both OS, corresponding to a cumulative incremental yield of 66% compared to the 1st-OS alone. Similarly, the number of cycles achieving ≥1 LBs increased from 225 to 506, corresponding to a cumulative incremental yield of +125% compared to the 1st-OS alone (**Figure 5A**). These findings were consistent throughout the study period. Overall, the cumulative live birth rate (cLBR) per started DuoStim cycle was 27% (N = 506/1896, 95% CI 25% to 29%; **Figure 5B**). When calculated only on concluded cycles, the cLBR was 28% (N = 506/1776, 95% CI 26% to 31%; **Figure 5C**), while the corresponding proportion per patient was 30.5% (N = 506/1656, 95% CI 28% to 33%). When considering concluded cycles after the 1st-OS and the 2nd-OS alone, the cLBR was 13% (225/1760; 95% CI 11% to 15%) and 17% (295/1756; 95% CI 15% to 19%; **Figure 5C**), respectively.

Interestingly, 26 patients obtained two LBs following DuoStim. Among them, 2 patients had both LBs from the 1st-OS, 13 patients had one from the 1st-OS and one from the 2nd-OS, and 11 patients had them both from the 2nd-OS. Notably, one patient achieved three live births from a single DuoStim cycle, with one derived from the 1st-OS and two from the 2nd-OS.

### Time to outcome and treatment decision patterns following DuoStim

**Figure 6:**
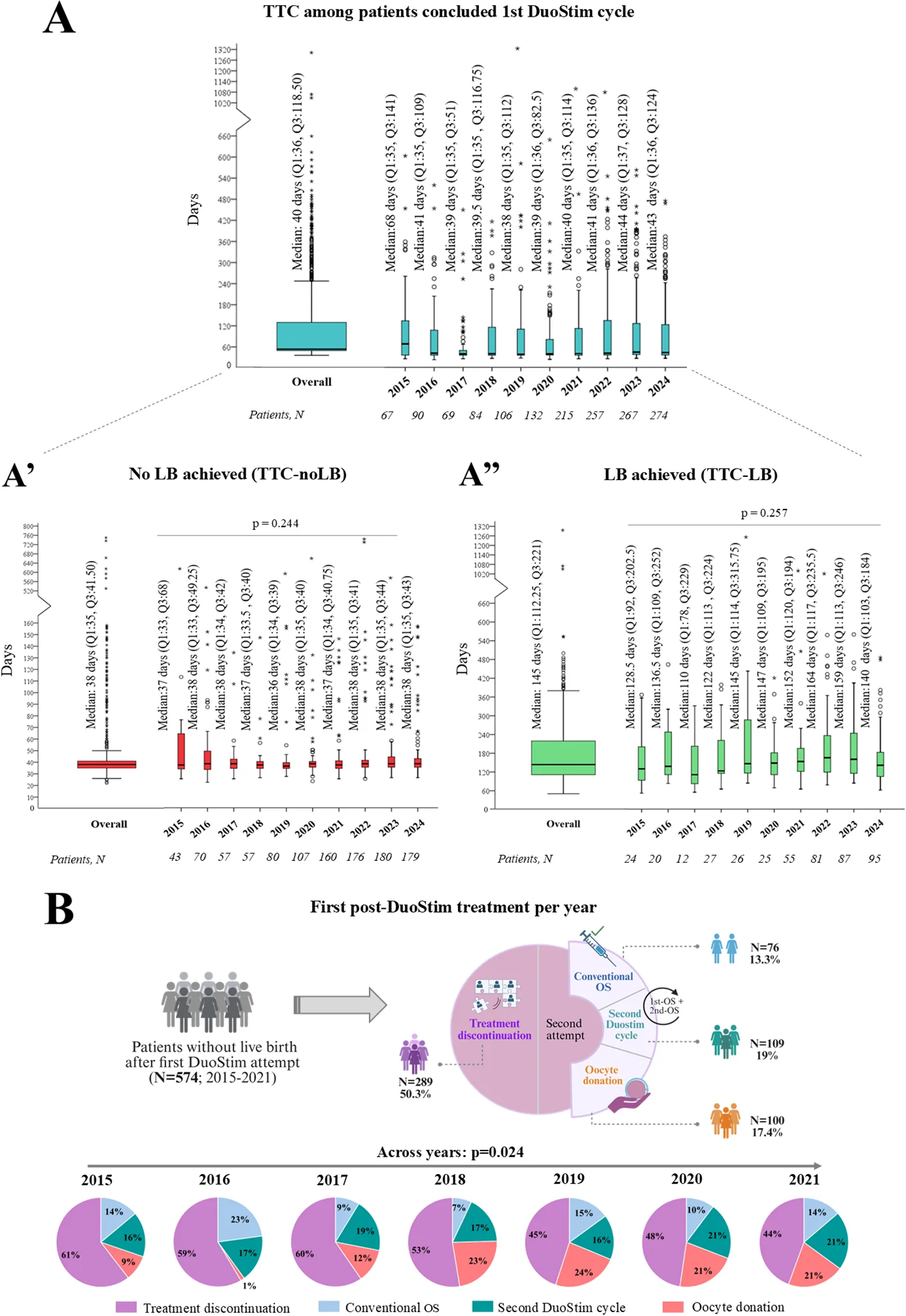
Time-related outcomes and treatment continuation patterns following DuoStim cycles. **(A)** Time to treatment conclusion (TTC), reported as median (Q1, Q3), with year-wise stratification shown. (A’) Time-to-treatment-conclusion without live birth (TTC-noLB), reported as median (Q1, Q3), with annual distribution across the study period. (A’’) Time-to-treatment-conclusion with live birth (TTC-LB), reported as median (Q1, Q3), stratified by year. **(B)** Treatment continuation patterns after the first DuoStim cycle, restricted to patients treated up to 2021, showing proportions of patients undergoing additional DuoStim cycles, conventional ovarian stimulation, oocyte donation, or discontinuing treatment.

The overall TTC was 40 days (Q1: 36, Q3: 118) and was calculated among 1561 patients who concluded a first DuoStim with or without a LB. A total of 95 patients with ongoing cycles were excluded from the analysis. When stratified by year, TTC values remained rather constant across the study period (**Figure 6A**). Specifically, TTC-noLB was 38 days (Q1: 35, Q3: 41.5) (p = 0.244; **Figure 6A’**), while the TTC-LB was 145 days (Q1: 112.25, Q3: 221), both stable (p = 0.257; **Figure 6A’’**). Following a first failed DuoStim cycle, patients could either discontinue the treatment or continue with further attempts, including a transition to a conventional OS, an additional DuoStim attempt, or a transition to an oocyte donation attempt. This analysis was restricted to 574 patients treated up to 2021 who did not achieve a LB. Overall, 109 patients (19%) underwent additional DuoStim cycles, 76 (13.3%) switched to conventional OS, and 100 (17.4%) transitioned to oocyte donation, while 289 (50.3%) discontinued treatment. Over time, treatment discontinuation progressively decreased, from 61% in 2015 to 44% in 2021 (p = 0.024), with a concomitant increase in the adoption of further treatment strategies, including a rise in oocyte donation from 9% to 21% over the same period. These changes likely reflect evolving clinical practice patterns over time. The proportions of patients undergoing additional DuoStim cycles and conventional OS remained relatively stable across years (**Figure 6B**). Univariate and multivariate logistic regression analyses were used to assess factors associated with treatment discontinuation. Collinearity among tested variables was excluded. Higher maternal age corresponded to increased odds of discontinuation (OR = 1.10, 95%CI: 1.03-1.17; p=0.002); significantly lower odds were instead observed in 2019–2021 compared with 2015 (OR 0.46, 95% CI 0.21–0.99, p = 0.047; OR 0.54, 95% CI 0.26–0.99, p = 0.050; OR 0.48, 95% CI 0.24–0.97, p = 0.040, respectively) (**Table 3**). The EPV was 36 (289 discontinuation events/8 model parameters), exceeding the recommended threshold of 10 and confirming adequate model stability. However, the AUC was 0.60 (95%CI 0.55-0.65), indicating limited discriminative ability of the model.

**Table 3:** Multivariate logistic regression analysis including factors found associated with the risk of treatment discontinuation after a first failed DuoStim cycle from univariate analyses.

Year	Multivariate-OR, 95%CI, p-value
2015	Control
2016	0.85, 95%CI: 0.39–1.87, p=0.691
2017	0.88, 95%CI: 0.39–2.00, p=0.768
2018	0.69, 95%CI: 0.31–1.55, p=0.364
2019	**0.46, 95%CI: 0.21–0.99, p=0.047**
2020	**0.54, 95%CI: 0.26–0.99, p=0.050**
2021	**0.48, 95%CI: 0.24–0.97, p=0.040**
**Maternal age**	**1.10, 95%CI: 1.03–1.17, p=0.002**

Tested variables that showed no association from univariate analyses were: cumulus oocyte complexes (COCs) retrieved after the first ovarian stimulation (1st-OS), number of previous cycles performed, presence of severe male factor, main cause of female infertility, duration of infertility, and time to conclusion of the first DuoStim.

Bold values indicate statistically significant differences.

## Discussion

In this ten-year retrospective analysis, we described the longitudinal implementation of DuoStim in a large cohort of poor-prognosis patients, focusing on its contribution to treatment efficiency, time-related outcomes, and patient management pathways in routine clinical practice. Rather than re-evaluating the biological rationale of the approach, which is already well-established, our findings provide insight into how this strategy performs when integrated in a routine multicycle IVF framework.

A key observation is the progressive adoption of DuoStim over time, reaching more than one-third of all cycles recently. This trend reflects increasing clinical confidence and supports its role as a concrete option in the management of time-sensitive patients. Importantly, this wider use occurred without changes in patient baseline characteristics or outcome metrics, suggesting that the strategy is reproducible across time. Consistent with previous evidence, the 2nd-OS contributed a higher number of oocytes and embryos compared with the 1st, while maintaining similar developmental competence. However, the clinical relevance of this finding lies less in the per-OS performance and more in the cumulative benefit. The 2nd-OS substantially increased the proportion of cycles achieving ≥1 euploid blastocyst and, most notably, more than doubled the number of cycles resulting in ≥1 LB compared with 1st-OS alone. These data reinforce the concept that, in poor-prognosis patients, success depends primarily on the ability to accumulate sufficient embryos rather than improving the efficiency of a single OS. In terms of safety, embryos derived from the 2nd-OS result in comparable clinical outcomes than those obtained after the 1st-OS. Moreover, although neonatal data were available only for a subset of cases and should be considered exploratory, no concerning patterns emerged. Both these results are in line with previous reports (19).

Beyond cumulative outcomes, one of the most clinically meaningful aspects emerging is the impact of DuoStim on time-related metrics. Notably, TTC-LB remained relatively stable over the years. Overall, one in three patients with a mean age above 40 obtained a LB through DuoStim, typically achieving this outcome within less than 5 months. For couples who did not conceive after a first DuoStim, instead, the TTC-noLB was around 40 days. This represents a relevant advantage in patients with limited reproductive time, in whom delays between consecutive attempts may compromise overall success. This temporal compression has relevant implications for patient management. By accelerating the completion of two OS, DuoStim enables early reassessment after failure and may support timely clinical decision-making.

In our cohort, a progressive reduction in treatment discontinuation rates over time was observed after a first failed DuoStim attempt, together with an increased adoption of further treatment strategies, including additional DuoStim cycles or transition to oocyte donation. These findings may reflect evolving clinical practice patterns and counselling approaches over time. Specifically, the rate of transition to oocyte donation more than doubled, rising from below 10% to over 20%. This occurred in the context of regulatory changes in Italy implemented shortly before the beginning of the study period. Within the evolving landscape of ART, there is a clear shift from a cycle-by-cycle evaluation of IVF success toward multicycle strategies aimed at maximizing overall treatment efficiency (36). In this framework, DuoStim represents a practical and well-established application of this paradigm, allowing two consecutive OS within the same ovarian cycle. More intensive approaches, such as triple stimulation (TriStim) (37) or quadruple stimulation (QuadriStim) (38), have been proposed with the aim of further increasing oocyte yield in highly selected population of poor prognosis patients; however, these strategies are currently supported by limited evidence. Therefore, among unconventional stimulation approaches, DuoStim represents a concrete strategy to improve treatment efficiency in time-sensitive patients. Its clinical relevance is further supported by its established use in urgent fertility preservation settings and its increasing recognition within international guidelines. In fact, ESHRE has revised its position on DuoStim, moving from a “for research only” stance toward broader acknowledgment of its clinical applicability in selected patient populations, including women with poor prognosis and limited reproductive time (39).

Beyond its clinical effectiveness, prior cost-effectiveness analyses from our group (17) suggest that concentrating two oocyte retrievals within a single ovarian cycle prevents the high risk of treatment discontinuation in highly poor prognosis patients, thereby increasing the take home baby rate per intention to treat despite a higher economic burden borne by couples. In this setting, the ability of DuoStim to secure a cumulative reproductive yield within a shortened timeframe translates into superior cost-effectiveness compared with a conventional cycle by cycle approach. However, these findings should be interpreted in the context of observational evidence and highly selected patient populations, in which treatment pathways are influenced by multiple clinical and organizational factors.

## Limitations

Some limitations of this study should be acknowledged. Its retrospective design and single-center setting may limit generalizability; however, they also ensured consistency in laboratory and clinical protocols, as well as expertise in managing this specific strategy. In addition, the absence of a concurrent control group undergoing sequential conventional OS precludes any direct comparison between strategies and does not allow the observed cumulative increase in oocyte, blastocyst, or euploid yield to be attributed to DuoStim itself. Therefore, our findings should be interpreted as descriptive of the cumulative outcomes achieved within this cohort rather than as evidence of superiority over sequential conventional OS. In this context, the BISTIM trial (15) and the randomized trial by Boudry et al. (16) represent important contributions to the field, particularly as they include comparator arms that are missing in our study design. However, direct extrapolation of their findings to the present work requires caution, as substantial differences exist in both biological rationale and clinical implementation. In particular, these trials differ from the DuoStim protocol applied at our center in several key aspects, such as the routine use of cleavage-stage embryo transfer, the implementation of fresh transfer in the first cycle, and the absence of a systematic blastocyst-stage culture combined with a freeze-all. Moreover, differences in oocyte handling and overall treatment objectives further limit comparability, as these studies are primarily designed around cycle- rather than treatment-based outcomes (40, 41).

Two main protocol transitions occurred during the study period, a shift from qPCR to NGS-based genetic testing and the introduction of PPOS in 2020, which could confound the temporal analyses. The former is unlikely to have affected the TTC. In fact, although NGS analytical turnaround time is longer than qPCR (42), both technologies were used at an external reference genetic laboratory, neutralizing temporal effects. Regarding PPOS protocol, although it shortened the duration of the 1st-OS by approximately 0.5 days compared to the antagonist-based DuoStim protocol (29), this difference is negligible. Accordingly, neither protocol transition is likely to have substantially influenced the temporal stability of TTC observed during the study period.

Lastly, neonatal follow-up data in our study were available only for a subset of newborns.

## Conclusions

Taken together, these findings support the interpretation of DuoStim not as a conventional cycle-based intervention, but rather as a time-sensitive, cumulative oocyte- and embryo-driven strategy. Its clinical value is therefore best appreciated in real-world cohorts of poor prognosis patients, where optimizing both efficiency and treatment continuity represents a critical unmet need. DuoStim represents a strategic and flexible approach within the multicycle paradigm for managing patients with limited reproductive time. Beyond its technical advantages, its use promotes a more realistic and transparent assessment of prognosis, facilitating alignment between clinical decision-making and patient expectations. In this sense, DuoStim contributes to a more structured and conscious approach to reproductive care in time-sensitive contexts. Looking ahead, the effectiveness of this strategy may be further enhanced through personalized medicine approaches supported by AI tools. The integration of AI-driven predictive models will enable more accurate patient profiling by combining clinical, hormonal, genetic, and dynamic response data. This may translate into a more targeted selection of candidates for DuoStim, allowing the identification of patients who are most likely to benefit from multistimulation strategies compared to conventional approaches, as well as into the optimization of stimulation protocols and a more reliable prediction of cumulative outcomes. Future research should further elucidate the biological mechanisms underlying the increased oocyte yield observed during the 2nd-OS and explore the potential extension of this strategy to additional indications, including PGT-M, PGT-SR, and severe male factor infertility.

## Data Availability

The original contributions presented in the study are included in the article/**Supplementary Material**. Further inquiries can be directed to the corresponding authors.

## Supplementary material

Supplementary Figure 1 Study flow diagram. Overview of patient inclusion, cycle progression, and analytical subsets, including concluded cycles, excluded ongoing cycles, and the discontinuation analysis cohort. *LB, Live Birth; TCC, Time to treatment conclusion; TTC-LB, Time to LB; TTC-noLB, Time to no LB; cLBR, cumulative live birth rate; SEBT, single euploid blastocyst transfer.*
